# The Effects of Different Warm-Up Protocols on Sprint Performance

**DOI:** 10.3390/sports14060250

**Published:** 2026-06-18

**Authors:** Frane Žuvela, Goran Kuvačić, Paula Matijašević, Josip Maleš, Hrvoje Ajman

**Affiliations:** 1 Faculty of Kinesiology, University of Split, 21000 Split, Croatia; frane.zuvela@kifst.eu (F.Ž.); goran.kuvacic@kifst.eu (G.K.); josipmales94@gmail.com (J.M.); 2 Research Group “Athletics and Kinesiological Development of Children and Youth”, Faculty of Kinesiology, University of Split, 21000 Split, Croatia; 3 Faculty of Kinesiology, University of Osijek, 31000 Osijek, Croatia; hrvoje.ajman@kifos.hr

**Keywords:** sprint performance, specific warm-up, repeated sprints, track and field

## Abstract

Background: Warm-up strategy is a key determinant of sprint performance, yet how different protocols influence performance across repeated sprint trials remains unclear, particularly regarding sex-specific responses. This study compared the acute effects of three warm-up protocols on repeated sprint performance in male and female athletes. Methods: Thirty-four male and 23 female athletes completed three warm-up protocols on separate occasions: a general long warm-up (GLW; 20 min), a long specific warm-up (LSW; 20 min), and a short specific warm-up (SSW; 10 min). After each protocol, participants performed three maximal 60 m sprints (T1, T2, T3), with split times recorded at 10 and 40 m. Sprint times were analysed using a three-way mixed-design ANOVA, with protocol and trial as within-subject factors and sex as the between-subject factor. Results: A significant protocol × sex interaction was observed at 40 m (F = 4.32, *p* = 0.016, ηp^2^ = 0.07) and 60 m (F = 4.08, *p* = 0.020, ηp^2^ = 0.07), but not at 10 m. Follow-up analyses showed no significant protocol differences in males. In females, LSW and SSW allowed faster sprint times than GLW at both 40 m and 60 m, while LSW and SSW did not differ from each other. The protocol × trial and protocol × trial × sex interactions were not significant. Conclusions: In this sample of student athletes, sprint-specific warm-ups allowed faster sprint performance than the general warm-up in females over 40 and 60 m, whereas no protocol differences were observed in males. These findings suggest that sex-specific responses to warm-up may be distance-dependent and should be interpreted in light of the heterogeneous sample and lack of menstrual-cycle control.

## 1. Introduction

Sprint performance is a decisive determinant of success in a wide range of sports, from athletic sprint disciplines to team competitions. In these disciplines, acceleration, the attainment of maximal velocity, and the ability to repeat high-intensity efforts directly influence the final competitive outcome [1,2]. Given that elite performances often differ by only hundredths of a second, even small acute improvements in athletes’ preparedness hold considerable practical value [3]. In this context, the warm-up represents the final modifiable step before performance and is regarded as a key component of competitive preparation, with the dual purpose of preparing the body for exertion and reducing the risk of injury [4,5].

The effects of warm-up on performance rely on a series of well-described short-term changes in the body, namely increases in muscle and body temperature, faster muscle contraction, improved oxygen supply, faster nerve signal conduction, and greater joint range of motion [5,6]. Evidence in recent studies also indicates that even a brief 10 min jogging warm-up can significantly increase skin temperature and also alter the mechanical properties of muscles such as the triceps surae, highlighting the relevance of warm-up as part of sprint and power performance training protocols [7]. When looking at sprinting specifically, warm-up protocols including ballistic exercises have been associated with better 100 m sprint performance in trained athletes [8]. Also, current evidence regarding different warm-up protocols favors ballistic and eccentric exercises over static stretching for overall better sprint performance [9,10].

In recent years, attention has increasingly shifted from thermal effects toward neuromuscular mechanisms, particularly toward the phenomenon known as post-activation performance enhancement (PAPE), a short-term increase in voluntary force and power following high-intensity effort [11,12]. The PAPE effect typically emerges within the first few minutes after the stimulus and is considered the main rationale for incorporating explosive exercises or sprint-like drills into the final phase of the warm-up prior to maximal efforts [10,13].

Although few would today question whether to warm up before sprint performance, the question of how to warm up remains open. Recent reviews indicate that combining low-intensity aerobic activity, dynamic mobility exercises, and sport-specific efforts produces better acute effects on sprint and jump performance than protocols dominated by static stretching or general activity alone [14,15]. Longer static stretching protocols repeatedly show the opposite effect, with acute reductions in maximal force and short-sprint performance [16,17], while excessive volumes of dynamic exercises can cause up to 2.6% impairment in sprint time due to accumulated fatigue [10,18]. The assumption that “longer” is also “better” has thus been called into question.

Among the many warm-up approaches used prior to maximal sprints [10] two have particularly stood out in the literature on sprint performance. The first adds plyometric exercises after a general warm-up to enhance neuromuscular activation [19]. The second uses sprinting itself as the final preparatory activity, based on the premise that a stimulus most similar to the target task ensures the greatest transfer of effect. The meta-analysis by Loturco et al. [20], however, showed that such protocols do not always lead to increased sprint velocity and that the outcome depends substantially on the preceding general warm-up, recovery, and participant characteristics. Direct comparisons of the two approaches within the same design remain rare.

The question becomes more complex when sex is considered. Although individual studies have examined the effects of warm-up in women [21,22], research in which the same warm-up protocols are directly compared between men and women remains rare. The available data point to different patterns of neuromuscular activation and time course of response to the stimulus between men and women [23,24]. On the other hand, the nature of sprint performance itself depends on the context. In track and field competition, an athlete performs only a single maximal sprint, so the choice of warm-up that enables the best performance in that single attempt is decisive. In the training process and team sports, by contrast, successive maximal efforts are routinely performed [25], so the question becomes which warm-up best maintains performance across a series of sprints. The empirical basis for answering both questions, particularly in mixed samples of men and women, remains limited. Taken together, the acute effects of warm-up specificity and duration on performance across repeated maximal sprint trials, and whether these effects differ between the sexes, have not been examined within a single study; this is the specific gap addressed by the present investigation.

The aim of this study was therefore to compare the acute effects of three warm-up protocols, a general long, a long specific, and a short specific warm-up based on progressive sprints, on 60 m sprint performance with split times at 10 m and 40 m, across three consecutive trials in male and female student athletes.

## 2. Materials and Methods

### 2.1. Participants

The initial sample comprised 41 men and 25 women (mean age 19.0 ± 0.7 years; sex-specific anthropometric data are reported in Table 1) representing a range of sporting backgrounds. According to the participant classification framework proposed by McKay et al. [26], all participants fell within Tier 1 (Recreationally Active) to Tier 2 (Trained/Developmental), indicating consistent participation in structured training without competing at an elite level. All reported a regular training routine of approximately five 1.5 h sessions per week, which included sprinting and running training. This combined sport and structured training background ensured that all participants had prior experience with maximal sprinting technique and with the dynamic mobility exercises used in the warm-up protocols.

**Figure 1:**
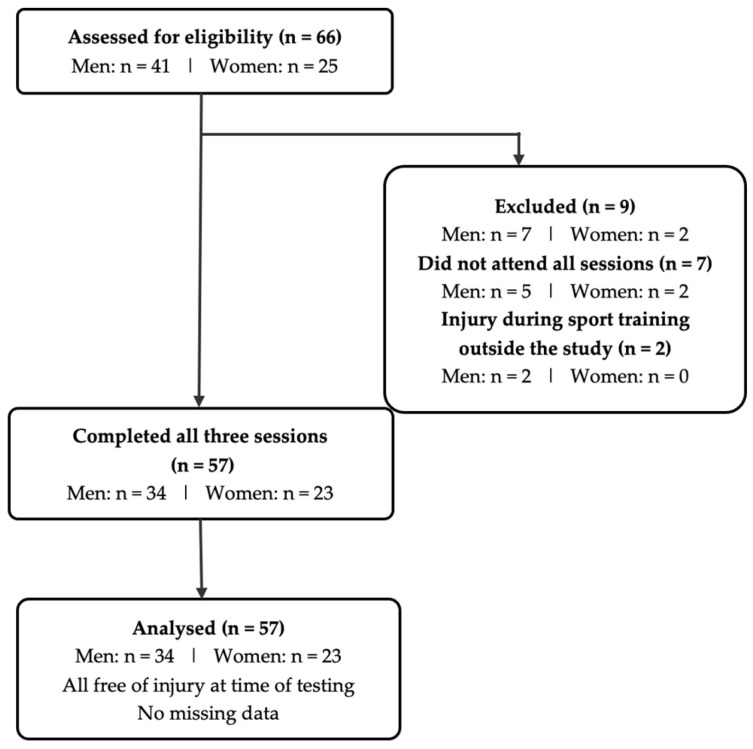
Flow diagram of participant enrolment, exclusion, and inclusion in the final analysed sample.

The exclusion criteria were: (i) acute or chronic musculoskeletal injuries, particularly of the lower extremities, within the preceding six months; (ii) diagnosed cardiovascular or respiratory conditions that could compromise safety or the execution of maximal effort; and (iii) incomplete participation in all warm-up sessions. Following the application of these criteria, nine participants (seven men and two women) were excluded from the final analysis: seven did not attend all three warm-up sessions, and two male participants sustained an injury during their regular sport training outside the study, which prevented them from completing the remaining sessions. This resulted in a final sample of 57 participants (34 men and 23 women), all of whom were free of injury at the time of testing; the participant flow is presented in Figure 1.

Participant anthropometric characteristics and the distribution of sport disciplines over the three calendar years preceding testing are summarised in Table 1. Among male participants, team-sport athletes were predominantly footballers (*n* = 12), while women in the team-sport group competed mainly in handball (*n* = 3) and volleyball (*n* = 2). Individual sports included dance (women *n* = 6), kickboxing, sport climbing, boxing, rugby, tennis, track and field, mixed martial arts, triathlon, rowing, middle-distance running, and equestrian. The fitness/strength category comprised gym training, general fitness, and street workout. The majority of participants (45 of 57; 79%) had been engaged in the same family of activity in each of the three preceding years, indicating a minimum continuous training age of three years.

Prior to enrolment, all participants were thoroughly briefed on the aims of the study, the testing procedures, the expected duration of participation, and the potential risks and benefits. Participants signed a written informed consent form, and participation was voluntary, with the possibility of withdrawing at any time without any consequences. The study was conducted in accordance with the Declaration of Helsinki and its subsequent revisions, and was approved by the Ethics Committee of the Faculty of Kinesiology, University of Split (approval number: 2181-205-02-05-26-019; date of approval: 20 April 2026).

### 2.2. Study Design

A within-subject experimental design with repeated measures was employed, in which each participant completed all three warm-up protocols across three separate test sessions, performing three maximal 60 m sprints in each session. Crossover design allowed for a direct comparison of the effects of individual protocols, with each participant serving as their own control. The order in which the three warm-up protocols were administered was randomised separately for each participant, so that no protocol was systematically associated with a particular session; this minimised systematic order effects. A one-week interval was maintained between each warm-up protocol. All measurements were conducted on the same outdoor athletics track, at the same time of day (±1 h) for each participant, to maintain comparable testing conditions across sessions. Testing was conducted during morning hours, with ambient air temperatures averaging 20–25 °C and no precipitation or strong wind recorded on any testing day. All participants wore standard running shoes, which, together with the use of the same outdoor athletics track for all sessions, ensured consistent footwear and surface conditions across protocols. No specific restrictions regarding training load, diet, or caffeine intake were imposed before the test sessions; however, the one-week interval between sessions and the within-subject crossover design, in which each participant served as their own control, limited the influence of any day-to-day variation in prior activity.

### 2.3. Warm-Up Protocols

The three warm-up protocols were based on the three-protocol design of van den Tillaar, Lerberg, and von Heimburg [27] and were chosen to contrast two sprint-specific preparation strategies against a common general warm-up used as a reference condition. The general long warm-up (GLW) and the short specific warm-up (SSW) followed the corresponding protocols of that study, whereas the long specific warm-up (LSW) was modified by replacing their progressive acceleration runs with a block of athletics running drills and plyometric jumps. The seven dynamic mobility exercises, as well as the order of components within each protocol, were kept constant across all three conditions.

*General Long Warm-Up (GLW):* The general long warm-up protocol consisted of 10 min of continuous running, during which participants subjectively rated the intensity between 4 and 6 on the Modified Borg Scale (Borg CR10 Scale). The Modified Borg Scale classifies the perception of exertion on a scale from 0 to 10, where 0 indicates no exertion and 10 represents maximal exertion [28]. Scherr et al. [29] established a strong association between perceived exertion and heart rate during treadmill running, supporting the use of the scale for intensity control. Participants were familiarised with the scale prior to the experiment in order to rate the intensity of their own exertion as accurately as possible. After the first 10 min of running, participants performed a block of seven dynamic mobility exercises in a fixed order, starting with the shoulder girdle and progressing downwards through the hip, knee, and ankle joints, with the aim of increasing the range of motion of each joint. Each exercise was performed in three sets of ten repetitions, giving a total of 210 repetitions, and the exercises were performed consecutively, with only the brief transition between exercises as recovery, so that heart rate did not decline substantially during this block. Depending on the tempo of execution, this dynamic block lasted approximately 5–7 min. The selection and ordering of these exercises were based on the protocol of van den Tillaar, Lerberg, and von Heimburg [27], and correct execution was ensured by the participants’ prior experience acquired through the curricular content of their study programme and their regular sporting practice. Upon completion of the dynamic block, participants resumed running at the same perceived intensity (4–6 on the Borg CR10 scale) for the remaining 3–5 min, so that the total duration of the protocol was 20 min. The GLW therefore comprised only low-to-moderate-intensity aerobic running and dynamic mobility exercises and, by design, contained no running drills, plyometric exercises, or accelerations, serving as the general, non-sprint-specific reference condition.

*Long Specific Warm-Up (LSW):* The first part of the long specific warm-up was identical to the GLW and comprised 10 min of running (intensity 4–6 on the Modified Borg Scale) followed by the same block of seven dynamic mobility exercises (three sets of ten repetitions each). After the dynamic block, the final segment of the warm-up consisted of sprint-specific running drills and plyometric jumps aimed at improving the biomechanical efficiency of sprinting and at acutely enhancing neuromuscular activation. This segment was performed in a fixed order of four exercises: A-skip, B-skip, bounding step, and alternating leg-to-leg jumps [30]. Each exercise was performed over a 20 m distance and was repeated twice, with the unhurried walk back to the starting line serving as the recovery between repetitions, giving a total drill volume of approximately 160 m. The running drills were executed with maximal attention to technical quality rather than to maximal locomotor speed, whereas the alternating leg-to-leg jumps constituted the plyometric component, comprising dynamic, stretch-shortening-cycle movements intended to induce post-activation potentiation and thereby acutely increase the muscle’s force-generating capacity [31,32]. The LSW thus combined aerobic running, dynamic mobility, sprint-specific drills, and plyometric jumps, but did not include maximal accelerations or sprints; its total duration was 20 min.

*Short Specific Warm-Up (SSW):* The short specific warm-up consisted of eight 60 m runs separated by 60 s of rest between efforts, for a total duration of approximately 10 min and a total running volume of 480 m. The first run was performed at a low, self-rated intensity of 4–6 on the Modified Borg Scale (Borg CR10 Scale), and the intensity of each subsequent run was increased in approximately equal increments so that the final runs were performed at maximal subjectively rated exertion. This graded progression was intended to raise muscle temperature and neuromuscular activation incrementally while limiting the early metabolic cost, with only the last runs approaching the demands of the test task. During each 60 s rest interval, participants performed one of the same seven dynamic mobility exercises used in the other two protocols [27], so that dynamic mobility content was equated across all conditions. The number, distance, and progression of the runs were adopted directly from the validated short specific warm-up of van den Tillaar et al. [27], on which the overall study design is based. This configuration was retained for three reasons: the 60 m distance reproduces the exact distance of the sprint test and therefore maximises the specificity of the conditioning stimulus; eight runs provide enough repetitions for a smooth, graded progression from submaximal to maximal intensity without an abrupt high-intensity onset; and the 60 s inter-run recovery keeps the protocol short (approximately 10 min), allowing its time-efficiency to be compared with that of the 20 min long protocols. Unlike the GLW and LSW, the SSW included no separate continuous aerobic block and no running drills or plyometric exercises; the progressive runs themselves served as both the aerobic and the sprint-specific conditioning component.

*Sprint Testing:* Upon completion of each warm-up protocol (20 min for the two long protocols and 10 min for the short protocol), participants were given three minutes of active recovery before performing three 60 m sprints, with three-minute intervals between sprints. This rest period was selected to allow for the near-complete restoration of the phosphagen energy system. Sprint times were recorded with a wireless single-beam photocell system (Witty, Microgate, Bolzano, Italy), which has a timing resolution of 0.125 ms and a manufacturer-declared impulse-transmission accuracy of ±0.4 ms. The photocells were mounted on tripods at hip height and positioned at 0, 10, 40, and 60 m along the running lane. Each sprint was performed from a stationary standing split-stance start, with the lead foot placed approximately 0.5 m behind the first gate to avoid premature triggering of the beam. Participants initiated each sprint in their own time, without an external signal, and timing was triggered automatically when the participant crossed the first photocell (0 m); split times were registered at 10 and 40 m and total time at 60 m.

Sprint running is traditionally divided into three main phases: acceleration, maintenance of maximal velocity, and deceleration [33]. Because the acceleration phase is itself complex and multidimensional, subsequent research has broken it down into several more specific sub-phases [34,35]. In the present study, the terminology proposed by Delecluse et al. [36] was adopted, in which the sprint is divided into initial acceleration (0–10 m), the attainment of maximal velocity (10–36 m), and the maintenance of maximal velocity (36–100 m). Accordingly, the analysis in the present study focused on the start and initial acceleration phase (0–10 m), the attainment of maximal velocity (10–40 m), and the maintenance of maximal velocity (40–60 m). Given that the factors influencing performance differ across sprint phases [37], success in one phase does not necessarily imply success in another, which justifies the separate analysis of split times over each of the examined distances.

### 2.4. Statistical Analysis

Data were processed using IBM SPSS Statistics (version 29.0, IBM Corp., Armonk, NY, USA). The normality of distribution was assessed with the Shapiro–Wilk test. Descriptive statistics included the arithmetic mean and standard deviation for each trial separately. The reliability of the three trials was estimated using the intraclass correlation coefficient (ICC) with a 95% confidence interval [38]. In addition, the typical error of measurement was calculated from the differences between consecutive trials and expressed in seconds and as a coefficient of variation (CV). Indicators of performance dynamics across trials included the mean times per trial, the percentage change from the first to the third trial calculated using the formula (T3 − T1)/T1 × 100, and the distribution of the best trial, which was tested with a χ^2^ goodness-of-fit test. The primary inferential analysis examined sprint performance across the three repeated sprint trials, separately for 10 m, 40 m, and 60 m. For each distance, a three-way mixed-design ANOVA was performed, with protocol and trial as within-subject factors and sex as the between-subject factor. This model tested the main effects of protocol, trial, and sex, as well as the protocol × sex, protocol × trial, trial × sex, and protocol × trial × sex interactions. If significant interaction effects were observed, follow-up simple-effects analyses were conducted as appropriate. For interactions involving protocol, pairwise comparisons of warm-up protocols were performed using estimated marginal means with Bonferroni correction. Sphericity was verified with Mauchly’s test, and in cases of violation, the Greenhouse-Geisser correction was applied. Effect size was estimated using partial eta-squared (η^2^), with values from 0.01 to 0.059 interpreted as small, from 0.06 to 0.139 as medium, and ≥0.14 as large effects [39]. The significance level was set at *p* < 0.05.

## 3. Results

The Shapiro–Wilk test confirmed the normality of distribution for most variables (*p* > 0.05), with the exception of SSW 60 m in men (*p* < 0.01). Descriptive parameters, reliability, and performance dynamics across the three trials are presented separately for male (Table 2) and female (Table 3) athletes. Intraclass correlation coefficients ranged from 0.75 to 0.97. The GLW protocol showed the highest reliability in both sexes (ICC = 0.91–0.97), while LSW showed the lowest (ICC = 0.75–0.95). Reliability was higher over the longer distances and in women compared with men. The typical error of measurement was similar between sexes, being 0.07, 0.12, and 0.18 s in men and 0.06, 0.12, and 0.17 s in women at 10, 40, and 60 m, respectively (CV 2.0–3.6%).

Performance dynamics across the three consecutive trials differed by sex. In men, performance remained relatively stable across trials under all protocols (change from T1 to T3 ranging from −0.64% to +2.31%); the smallest changes occurred under GLW (+0.18% to +0.40%) and the largest under LSW at 40 and 60 m (+2.16% and +2.31%). In women, performance declined progressively across trials under all protocols, with the most pronounced slowing under GLW (+2.18% to +2.48%) and the smallest decline under SSW (+0.48% to +1.39%).

The distribution of individual best trials also differed by sex. In men, the proportion of participants achieving their fastest sprint in T1, T2, or T3 did not differ significantly from chance at any distance or protocol (all χ^2^ *p* > 0.05). In women, by contrast, the majority achieved their best sprint in the first trial, significantly so for SSW at 40 and 60 m (χ^2^ = 17.04 and 18.09, both *p* = 0.001) and for GLW at all distances (10 m: χ^2^ = 8.43, *p* = 0.02; 40 m: χ^2^ = 10.78, *p* = 0.01; 60 m: χ^2^ = 25.13, *p* = 0.001), indicating a progressive decline in performance across repeated sprints that was most pronounced under the general warm-up.

A three-way mixed-design ANOVA examined the effects of protocol, trial, and sex on sprint performance at 10, 40, and 60 m (Table 4). A significant main effect of protocol was found at all distances, while the main effect of trial was significant only at 40 and 60 m. The protocol × sex interaction was significant at 40 and 60 m, but not at 10 m, indicating sex-specific protocol effects over the longer sprint distances. The protocol × trial and protocol × trial × sex interactions were not significant at any distance.

**Figure 2:**
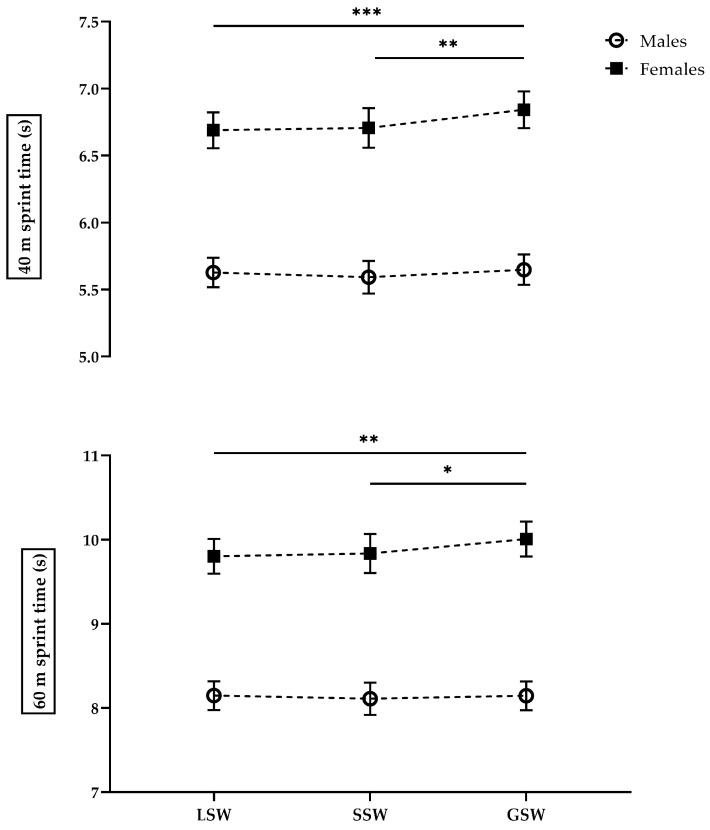
Protocol × sex interaction for 40 m and 60 m sprint performance. Values represent estimated marginal means averaged across the three sprint trials. Error bars indicate SE; LSW = long specific warm-up; SSW = short specific warm-up; GLW = general long warm-up. * *p* < 0.05; ** *p* < 0.01; *** *p* < 0.001.

Follow-up simple-effects analyses of the significant protocol × sex interactions are presented in Figure 2. In males, no significant differences among warm-up protocols were observed at either 40 or 60 m. In females, however, both sprint-specific warm-ups allowed faster sprint times than the general long warm-up at 40 and 60 m, while LSW and SSW did not differ significantly from each other. These results indicate that the significant protocol × sex interaction was primarily driven by protocol-related differences in females, particularly the slower performance following GLW compared with the two sprint-specific protocols.

## 4. Discussion

The aim of this study was to compare the acute effects of three warm-up protocols on sprint performance at 10, 40, and 60 m across three repeated maximal sprints in male and female athletes. The main finding was that the effect of warm-up protocol differed by sex at 40 and 60 m, but not at 10 m. Follow-up analyses showed that this interaction was driven by significant protocol-related differences in females, in whom both sprint-specific protocols allowed faster sprint times than the general long warm-up. In males, no significant differences among protocols were observed at 40 or 60 m. The protocol × trial and protocol × trial × sex interactions were not significant, indicating that protocol-related differences did not change substantially across the three sprint trials. Descriptively, however, performance dynamics differed between protocols, particularly in females, where GLW showed the greatest decline and SSW the smallest decline across repeated sprints.

Testing the protocol × sex interaction directly places the central role of sex on a firm statistical footing. The protocol × sex interaction was significant at 40 and 60 m but not at 10 m, and follow-up simple-effects analyses confirmed a clear protocol effect in women, in whom LSW and SSW allowed significantly faster sprints than GLW, whereas no protocol effect was present in men at any distance (full statistics are reported in the Results and in the corresponding table). This justifies the sex-stratified discussion that follows. The absence of an interaction at 10 m deserves separate consideration. Sprinting comprises phases with distinct physiological and biomechanical demands [35,40]. The first 10 m represents the initial acceleration phase, the performance of which is governed primarily by the ability to produce and orient horizontal ground-reaction force at low running velocities. This horizontal force application is the strongest mechanical determinant of short-sprint acceleration, more important than the absolute magnitude of the force produced [41,42], and corresponds to the force-dominant region of the force-velocity continuum [43]. Mechanically, the decisive feature of this phase is the production of a large propulsive, horizontally oriented ground-reaction force throughout acceleration [44]. Two features of this phase reduce the scope for the specific protocols to differentiate performance at 10 m. First, force-production capacity is acutely enhanced by the temperature and muscle-stiffness changes that any adequate warm-up produces; Meerits et al. [7] showed that even 10 min of jogging acutely increases tone and stiffness of the triceps surae and elevates skin temperature in track-and-field athletes, and because all three protocols shared a warming and mobility component, this force-production substrate was prepared similarly across conditions. Second, the orientation of force application reflects a technical ability that develops with training [41], and is therefore unlikely to be substantially altered by the acute type of warm-up. By contrast, the maximal-velocity phase rests on different qualities. Here performance depends on the ability to apply a large, predominantly vertical ground-reaction force [44], which in turn requires a high rate of force development [45]. These are precisely the qualities trained by the sprint drills and plyometric jumps of LSW and by the progressive sprints of SSW. Comparable conditioning stimuli have been shown to acutely raise maximal sprint velocity and step frequency [46], consistent with the principle that warm-up transfer is greatest when the conditioning activity resembles the target task [4,5,10]. Their incremental advantage over GLW was therefore greatest at 40 and 60 m. The between-sex difference in response thus emerges precisely where the specific protocols add value beyond GLW. The somewhat lower measurement reliability at 10 m may also reduce the statistical power to detect a moderate interaction at that distance [47].

The absence of statistically significant differences between protocols at the level of the best result in male athletes is consistent with the findings of several recent studies. The meta-analysis by Loturco et al. [20] showed that various specific stimuli incorporated into the warm-up often fail to acutely increase sprint velocity in competitive sprinters, and Silva et al. [19] likewise concluded in a systematic review that PAPE protocols in team sports produce small and variable effects, often below the threshold of practical significance. Part of the literature, however, reports the opposite pattern. Van den Tillaar et al. [27], employing a similar three-protocol design (LGW, LSW, SSW) on which our protocols are also based, reported significantly faster sprint times after both specific warm-ups in 12 experienced male soccer players. In the same direction, several studies have demonstrated significant improvements in sprint performance in male athletes following sprint-specific or plyometric warm-up protocols, with improvements ranging from 1.6 to 3% [48,49,50]. The difference relative to our results in male athletes most likely reflects the heterogeneous composition of our sample (student athletes from various sports) compared with the homogeneous specialised populations, in which the same conditioning stimuli may produce more consistent acute effects. Consistent with this interpretation, the meta-analysis by Wilson et al. [51] found that the power-augmenting response to a conditioning activity increases with training status, being substantially larger in athletes (ES = 0.81) than in trained (0.29) or untrained (0.14) individuals. The modest effects in our non-specialised cohort are consistent with this gradient. The reason for these differences in findings likely lies in the training level of the participants, the type and intensity of the activity, and the duration of recovery between the stimulus and performance [20,52]. Specifically, studies reporting positive effects most often involved homogeneous samples of elite soccer players or sprinters, whereas the sample in the present study consisted of student athletes from various sports, a pattern theoretically consistent with the idea that sprint performance in trained male athletes is a highly stable physical ability with limited room for acute potentiation [2,11]. In addition, slightly lower reliability values in men point to greater intra-individual variability that may mask small systematic differences between protocols [53]. Of note, the descriptive analysis clearly showed that LSW yielded the fastest sprints in the first trial at 40 m and 60 m, while in subsequent trials the differences between protocols levelled out. This pattern suggests that the transient performance-enhancing effect of sprint drills and plyometric exercises is also present in male athletes, but is transient and of a magnitude insufficient to reach statistical significance at the level of the best result, which is in line with the findings of Creekmur et al. [31], who likewise observed small and inconsistent effects of plyometric warm-up on 20 m and 40 m sprint performance in male athletes. Accordingly, we interpret the lack of statistical significance in men not as an absence of effect but as an effect of insufficient magnitude under conditions of heterogeneous sampling and limited statistical power [47].

In contrast to the results in men, female athletes showed a markedly greater response to both specific warm-up protocols, with differences of approximately 0.2 s between LSW and GLW at 40 m and 60 m, which in sprint disciplines often determines the final ranking in competition [2,3]. In the context of sprinting specifically, PAPE is described in the literature as a transient, conditioning induced enhancement of voluntary force and power and of the capacity for rapid force production, which translates into improved performance in subsequent explosive tasks such as sprinting [11,12,54]. The protocol effects observed in the present study are therefore interpreted within this established framework. The results are consistent with the recent study by Zylberberg et al. [22], who reported acute benefits of a potentiating warm-up protocol in female soccer players, and with the findings of Matusiński et al. [21] in competitive female sprinters. Nevertheless, part of the literature presents a different picture. Chaves Lucas et al. [55] found no significant differences between three different warm-up protocols on 20 m sprint performance in trained female futsal athletes. Such differences likely arise from the interplay between training level, type of stimulus, duration of recovery [13,52,56]. One possible reason for the greater response in our sample is the lower absolute level of sprint performance in women, which theoretically leaves more room for acute potentiation [13,53]. In addition, women show faster metabolic recovery between consecutive sprints, smaller absolute decline in mechanical work, and different patterns of neuromuscular activation compared with men [23,56,57], which makes them more suitable candidates for protocols based on repeated efforts. These characteristics are consistent with the broader PAPE construct described by Blazevich and Babault [12], which encompasses not only classical regulatory light chain phosphorylation but also temperature, fluid shifts, muscle compliance, and neural drive. These components can collectively favour a more pronounced acute response to specific warm-up stimuli in female athletes. Koźlenia and Domaradzki [24] showed that in trained adults the peak of the PAPE effect occurs as early as the third minute after the stimulus, although in women this peak is short-lived and rapidly declines, whereas in men it persists for several additional minutes. This temporal pattern coincides with the timeframe of our testing and may explain the pronounced effect of LSW in the first trial in our female sample. Because our measurements were performed exactly at the 3 min mark, this window aligns almost ideally with the PAPE peak in women, while in men part of the potential response may have been temporally missed by the recovery interval, plausibly contributing to the observed sex asymmetry. From an applied perspective, the present findings suggest that, in this sample, females may benefit more from sprint-specific warm-ups than males over 40 and 60 m, where the protocol × sex interaction reached significance. However, this interpretation should be considered in light of the heterogeneous sporting background of the participants, the absence of menstrual-cycle and hormonal-contraceptive control, and the fact that baseline sprint level was not included as a covariate.

A particularly interesting finding emerges from the descriptive trial-by-trial analysis in females. LSW produced faster times immediately after the warm-up, whereas SSW showed the smallest decline from T1 to T3, especially at 40 m and 60 m. However, because the protocol × trial and protocol × trial × sex interactions were not significant, these patterns should be interpreted descriptively rather than as evidence of a statistically confirmed delayed protocol effect. Mechanistically, LSW combines an aerobic and mobilisation component with sprint drills and plyometric exercises of relatively low energy cost, which may allow an immediate performance response without substantial fatigue accumulation [31,32]. In contrast, SSW may represent both a potentiating and fatiguing stimulus because it includes eight progressive 60 m runs; therefore, its practical value may lie in providing a time-efficient, sprint-specific preparation strategy rather than in producing a statistically proven progressive benefit across trials in the present analysis. This dual role of SSW has been described in the work of Tillin and Bishop [54], in which the net performance outcome after a high-intensity conditioning stimulus depends on the balance between fatigue and potentiation. Similar fatigue–potentiation interactions have also been reported after sprint-specific warm-up strategies in soccer players [49]. Consistent with this, the meta-analysis by Wilson et al. [51] confirmed that more intensive conditioning activities generate both larger potentiation and larger residual fatigue, which dissipate at different rates during recovery. In the present study, the 3 min interval between SSW and the first test sprint falls within the early post-conditioning window in which fatigue can still dominate over potentiation for a stimulus of this magnitude; by T2 and T3 (approximately 5 to 7 min after SSW), fatigue has likely dissipated, allowing the net performance-enhancing effect to emerge. This interpretation suggests that, when SSW is used before a single decisive maximal effort, more than 3 min of passive recovery before performance may be advisable. Despite these distinct mechanistic pathways, the statistical equivalence of LSW and SSW across all comparisons points to a common final pathway of acute readiness, in which different conditioning inputs (drill-based plus plyometric activation versus progressive maximal sprinting) appear to converge on a similar level of neuromuscular preparation for the subsequent sprint task. This implies that the acute readiness response is driven by warm-up specificity in a broad sense rather than by any single canonical recipe, and that practitioners can adapt the warm-up to time, environmental, and athlete-profile constraints without sacrificing efficacy [15]. From a practical perspective, LSW appears to be optimal when an athlete performs a single decisive maximal effort, as it ensures immediate readiness, whereas SSW offers more stable performance across consecutive sprints, making it useful in the context of training or sports involving repeated sprints [19]. It should also be noted that although the omnibus effect of warm-up was large (partial η^2^ up to 0.53), the significant pairwise differences corresponded to small-to-medium standardised effects (Cohen’s d ≈ 0.3 to 0.6; absolute differences ≈ 0.06 to 0.23 s). The largest differences in women at 40 and 60 m exceed the typical error of measurement, whereas the smallest differences at 10 m approach it, justifying caution in interpreting differences of that magnitude.

These findings have clear implications for structuring warm-up in practice, particularly in female athletes, in whom the choice of protocol represents a significant factor of acute sprint performance. The general warm-up without a sprint-specific component consistently yielded slower sprints, which is in line with the recommendations of recent reviews emphasising that the final, sport-specific part of the warm-up should be a central component of preparation for maximal efforts [10,15]. When the goal is a single decisive maximal effort, LSW appears to be the optimal choice due to immediate performance enhancement, while SSW represents an equally effective but shorter alternative in the context of training or sports involving repeated sprints. In male athletes the differences are considerably less pronounced, but the descriptive patterns follow a similar logic: for a single maximal effort, both specific protocols allow faster sprints than the general one, whereas for repeated sprints SSW shows consistently faster results with substantially shorter duration. The fact that the 10 min SSW achieved comparable results to the 20 min LSW carries clear operational value, particularly in team sports where the warm-up must be aligned with the tactical and technical components of preparation [5,19]. The benefits of a warm-up are, moreover, time-sensitive: in semi-professional soccer players, Kyranoudis et al. [58] found that a 15 min transition period after the warm-up significantly impaired jump performance and tended to reduce sprint performance, indicating that warm-up-induced readiness decays over time. This reinforces the practical value of a short, sprint-based protocol such as the SSW, which can be completed close to the moment of performance.

### Limitations

This study has several limitations that should be taken into account when interpreting the findings. The sample consisted of student athletes from various disciplines rather than a homogeneous group of specialised sprinters or players from a single team sport, which limits the generalisability of the findings to the elite sprint population. In addition, only three consecutive sprints with a relatively long recovery interval (3 min) were tested, so the question remains whether longer protocols or shorter recovery intervals would yield a different picture of accumulated fatigue [25]. Future research should include longer series of repeated sprints, more homogeneous samples of competitive athletes, and direct measurement of neuromuscular parameters in order to further clarify the mechanisms of sex-specific responses to different warm-up protocols, and it would also be valuable to compare the effects of LSW and SSW in a real competitive context. In addition, menstrual-cycle phase and hormonal status were not recorded or controlled in the female participants, who also differed in training background and baseline sprint level; because these factors may influence the acute response to warm-up, the sex-specific findings should be considered preliminary and would benefit from confirmation in more homogeneous, controlled samples. Finally, because neuromuscular fatigue and potentiation were not measured directly (for example, by electromyography or evoked twitch responses), the relative contributions of potentiation, residual fatigue, temperature, learning, and pacing to the observed responses, and in particular to the descriptive trial-by-trial pattern observed after SSW, could only be inferred rather than quantified. Accordingly, the terms post-activation performance enhancement (PAPE) and potentiation are used in this manuscript in a descriptive sense, to denote acute improvements in sprint performance, rather than as confirmed physiological mechanisms.

## 5. Conclusions

This study showed that the acute effects of warm-up protocol on sprint performance differed by sex over the longer sprint distances. A significant protocol × sex interaction was observed at 40 and 60 m, but not at 10 m. In male athletes, no significant protocol differences were observed at 40 or 60 m, although descriptive patterns suggest that sprint-specific warm-ups may still be practically relevant in some contexts. In female athletes, a clear and practically relevant advantage of LSW and SSW over GLW was established, with large effect sizes at 40 m and 60 m. The two specific protocols differed in their temporal dynamics, as LSW produced an immediate potentiation visible already in the first trial, whereas SSW exhibited a delayed effect that became pronounced in the second and third trials.

From an applied perspective, in female athletes LSW stands out as the optimal choice prior to a single maximal sprint, while SSW represents a time-efficient alternative for training or sports involving repeated sprints. The equal effectiveness of the 10 min SSW and the 20 min LSW carries clear operational value in everyday coaching practice, particularly when warm-up time must be aligned with the tactical and technical components of preparation.

## Figures and Tables

**Table 1:** Anthropometric and sport-participation characteristics of participants.

Variable	Men	Women
Age (years) (M ± SD)	19.0 ± 0.7	19.0 ± 0.7
Body mass (kg) (M ± SD)	76.21 ± 9.96	65.67 ± 9.23
Body height (m) (M ± SD)	1.81 ± 0.06	1.70 ± 0.06
Team sports, *n* (%)	16 (47.1)	6 (26.1)
Individual sports, *n* (%)	12 (35.3)	9 (39.1)
Fitness/strength training, *n* (%)	6 (17.6)	8 (34.8)
Continuous training ≥ 3 years, *n* (%)	28 (82.4)	17 (73.9)

**Table 2:** Descriptive parameters, reliability and performance dynamics across three sprint trials in male athletes (*n* = 34).

W-Up	Distance	T1	T2	T3	T1→T3	Trend	ICC
		M ± SD	M ± SD	M ± SD			(95% CI)
LSW	10 m	1.82 ± 0.11	1.82 ± 0.10	1.83 ± 0.12	+1.04	↑	0.75 (0.56–0.87)
	40 m	5.57 ± 0.28	5.63 ± 0.26	5.69 ± 0.38	+2.16	↑↑	0.88 (0.78–0.94)
	60 m	8.07 ± 0.43	8.12 ± 0.42	8.25 ± 0.57	+2.31	↑↑	0.87 (0.77–0.93)
SSW	10 m	1.81 ± 0.14	1.81 ± 0.14	1.79 ± 0.12	−0.39	↓	0.89 (0.80–0.94)
	40 m	5.56 ± 0.27	5.61 ± 0.33	5.61 ± 0.33	+0.92	↑	0.92 (0.85–0.95)
	60 m	8.06 ± 0.51	8.10 ± 0.49	8.17 ± 0.55	+1.27	↑	0.93 (0.88–0.97)
GLW	10 m	1.85 ± 0.15	1.83 ± 0.15	1.84 ± 0.13	−0.64	↓↓	0.91 (0.85–0.95)
	40 m	5.66 ± 0.26	5.61 ± 0.29	5.67 ± 0.29	+0.18	→	0.94 (0.90–0.97)
	60 m	8.16 ± 0.42	8.08 ± 0.42	8.19 ± 0.45	+0.40	→	0.95 (0.92–0.98)

Legend: T1→T3 (%): percentage change (+ = slowing). Trend: → stable; ↑ slowing; ↑↑ marked; ↑↑↑ pronounced; ↓ acceleration; ↓↓ pronounced; LSW = Long Specific Warm-up; SSW = Short Specific Warm-up; GLW = General Long Warm-up.

**Table 3:** Descriptive parameters, reliability and performance dynamics across three sprint trials in female athletes (*n* = 23).

W-Up	Distance	T1	T2	T3	T1→T3	Trend	ICC
		M ± SD	M ± SD	M ± SD	(%)		(95% CI)
LSW	10 m	2.09 ± 0.11	2.10 ± 0.13	2.12 ± 0.16	+1.53	↑↑	0.86 (0.71–0.93)
	40 m	6.62 ± 0.34	6.71 ± 0.41	6.74 ± 0.46	+1.77	↑↑	0.94 (0.88–0.97)
	60 m	9.70 ± 0.50	9.82 ± 0.61	9.90 ± 0.67	+2.05	↑↑	0.95 (0.90–0.98)
SSW	10 m	2.11 ± 0.16	2.12 ± 0.16	2.11 ± 0.14	+0.48	→	0.93 (0.85–0.97)
	40 m	6.65 ± 0.44	6.74 ± 0.46	6.73 ± 0.46	+1.07	↑	0.97 (0.93–0.98)
	60 m	9.76 ± 0.65	9.86 ± 0.56	9.89 ± 0.67	+1.39	↑	0.96 (0.93–0.98)
GLW	10 m	2.13 ± 0.13	2.16 ± 0.15	2.19 ± 0.17	+2.48	↑↑↑	0.93 (0.86–0.97)
	40 m	6.77 ± 0.40	6.85 ± 0.41	6.91 ± 0.42	+2.18	↑↑↑	0.97 (0.92–0.99)
	60 m	9.88 ± 0.60	10.03 ± 0.61	10.12 ± 0.62	+2.45	↑↑↑	0.96 (0.90–0.97)

Legend: T1→T3 (%): percentage change (+ = slowing). Trend: → stable; ↑ slowing; ↑↑ marked; ↑↑↑ pronounced; ↓ acceleration; ↓↓ pronounced; LSW = Long Specific Warm-up; SSW = Short Specific Warm-up; GLW = General Long Warm-up.

**Table 4:** Three-way mixed-design ANOVA: within- and between-subject main effects and interactions for sprint times at 10, 40, and 60 m.

	df	F	*p*	ηp^2^
*10 m*				
Protocol	2, 110	7.23	0.001	0.12
Protocol × Sex	2, 110	1.67	0.192	0.03
Trial	2, 110	1.90	0.155	0.03
Trial × Sex	2, 110	1.93	0.150	0.03
Protocol × Trial	3.36, 184.71	0.87	0.468	0.02
Protocol × Trial × Sex	3.36, 184.71	0.69	0.573	0.01
Sex (between-subjects)	1, 55	103.59	<0.001	0.65
***40 m***				
Protocol	2, 110	10.99	<0.001	0.17
Protocol × Sex	2, 110	4.32	0.016	0.07
Trial	1.76, 97.06	14.45	<0.001	0.21
Trial × Sex	1.76, 97.06	2.18	0.125	0.04
Protocol × Trial	3.04, 167.15	1.82	0.144	0.03
Protocol × Trial × Sex	3.04, 167.15	1.32	0.270	0.02
Sex (between-subjects)	1, 55	169.16	<0.001	0.76
***60 m***				
Protocol	2, 110	5.12	0.007	0.09
Protocol × Sex	2, 110	4.08	0.020	0.07
Trial	1.70, 93.37	18.96	<0.001	0.26
Trial × Sex	1.70, 93.37	3.29	0.049	0.06
Protocol × Trial	2.67, 147.14	0.78	0.493	0.01
Protocol × Trial × Sex	2.67, 147.14	1.27	0.288	0.02
Sex (between-subjects)	1, 55	173.31	<0.001	0.76

Legend: F = F statistic; df = degrees of freedom; *p* = significance; ηp^2^ = partial eta-squared.

## Data Availability

The data presented in this study are available on request from the corresponding author. The data are not publicly available due to privacy restrictions concerning the participating athletes.

## Abbreviations

The following abbreviations are used in this manuscript:

GLW	General Long Warm-up
LSW	Long Specific Warm-up
SSW	Short Specific Warm-up
PAPE	Post-Activation Performance Enhancement
